# BTG1 Overexpression Might Promote Invasion and Metastasis of Colorectal Cancer *via* Decreasing Adhesion and Inducing Epithelial–Mesenchymal Transition

**DOI:** 10.3389/fonc.2020.598192

**Published:** 2020-11-27

**Authors:** Shuang Zhao, Hang Xue, Chang-lai Hao, Hua-mao Jiang, Hua-chuan Zheng

**Affiliations:** ^1^ Department of Oncology and Experimental Center, The Affiliated Hospital of Chengde Medical University, Chengde, China; ^2^ Department of Hematology, The Affiliated Hospital of Chengde Medical University, Chengde, China; ^3^ Department of Urology, The First Affiliated Hospital of Jinzhou Medical University, Jinzhou, China

**Keywords:** colorectal cancer, *BTG1*, adhesion, invasion, metastasis, prognosis

## Abstract

BTG (B-cell translocation gene) could inhibit cell proliferation, metastasis, and angiogenesis and regulate cell cycle progression and differentiation in a variety of cancer cell types. To clarify the role of *BTG1* in invasion and metastasis, its expression was compared with the clinicopathological parameters of colorectal cancer by bioinformatics and immunohistochemical analyses. We also overexpressed BTG1 in HCT-15 cells and examined its effects on adhesion, migration, and metastasis with their related molecules screened. BTG1 mRNA expression was negatively correlated with its promoter methylation in colorectal cancer (*P* < 0.05). Among them, cg08832851 and cg05819371 hypermethylation and mRNA expression of *BTG1* were positively related with poor prognosis of the colorectal cancer patients (*P* < 0.05). BTG1 expression was found to positively correlate with depth of invasion, venous invasion, lymph node metastasis, distant metastasis, and TNM staging of colorectal cancer (*P* < 0.05) but negatively with serum levels of CEA and CA19-9 (*P* < 0.05). According to the TCGA database, BTG1 mRNA expression was lower in well-, moderately, and poorly differentiated than mucinous adenocarcinomas and positively correlated with ras or BRAF mutation (*P* < 0.05). Kaplan–Meier analysis showed the negative correlation between *BTG1* mRNA expression and overall survival rate of all cancer patients (*P* < 0.05). BTG1 overexpression weakened adhesion and strengthened migration and invasion of HCT-15 cells (*P* < 0.05). There was E-cadherin hypoexpression, N-cadherin and MMP-9 hyperexpression, *Zeb1* and *Vimentin* mRNA overexpression, a high expression of *CEA* mRNA and protein, and a strong secretion of CEA in *BTG1* transfectants, compared with the control or mock. It was suggested that BTG1 expression might promote invasion and metastasis by decreasing adhesion, and inducing epithelial–mesenchymal transition.

## Introduction

Colorectal cancer is one of the most common cancers in the world, accounting for nearly 10% of new cases of all cancers. Its invasion and metastasis greatly determine clinical outcome, survival time, and quality of the patients (1). Therefore, it is important to find out the molecular mechanisms and related targets for the invasion and metastasis of colorectal cancers.

As a tumor suppressor, BTG1 overexpression is observed in the G_0_/G_1_ phases of the cell cycle and its down-regulation once cell progression through G_1_. It might inhibit cell proliferation and cell cycle progression (2). BTG1 induced the apoptosis of NIH 3T3 cells, was localized to the cells with DNA fragmentation and nuclear condensation, and contributed to antisense Bcl-2-mediated cytotoxic effects, indicating its apoptotic induction *via* mitochondrial pathway (3, 4). BTG1 activation was found to regress oxidative stress and cytokine expression in macrophages by inhibiting AP-1 and NF-*к*B (3). Reportedly, BTG1 overexpression might promote tube formation and cell migration of endothelial cells during angiogenesis (5). BTG1 could interact with nuclear receptor TR*α*, myogenic factor MyoD, carbon catabolite repressor protein-associative factor 1, and arginine methyltransferase 1 (6–8).

miR-4295 overexpression was found to significantly promote proliferation, colony formation, and migration of bladder cancer cells *via* directly targeting BTG1 at its 3′-UTR (9). BTG1 could reverse the inhibition of autophagy induced by miR-22 (10). BTG1 functioned as a direct target of miR-330-3p and miR-27a-3p in hepatocellular carcinoma and ovarian cancer cells, respectively and thereby weakened cell viability, migration, and invasion and promoted cell apoptosis (11, 12). BTG1 was shown to prevent antigen from inducing molecular features of *in vitro* allergic reactions as a direct target of miR-183-5p (13).

Our previous study demonstrated that BTG1 overexpression inhibited proliferation, tumor growth or lung metastasis, induced differentiation, autophagy, apoptosis, or mediated chemosensitivity in colorectal and gastric cancer cells (14, 15). Su et al. (16) reported that BTG1 overexpression triggered G_1_/S phase cell cycle arrest and increased apoptosis in HCT-116 cells *via* ERK/MEK pathway. BTG1 induced Beclin-1-dependent autophagy and weakened *β*-catenin pathway in colorectal cancer cells (14). In ovarian cancer, BTG1 expression caused a low growth rate, high cisplatin sensitivity, G_1_ arrest, apoptosis and decreased migration and invasion by down-regulating the expression of PI3K, PKB, Bcl-xL, survivin, VEGF, and MMP-2. There was higher expression of *BTG1* mRNA in normal tissue than that in ovarian cancer tissue, and in benign tumors than in cancer tissue. *BTG1* mRNA expression was negatively correlated with FIGO staging of ovarian cancer (17). In the present study, we mainly investigated the roles of BTG1 expression on progression of colorectal cancers.

## Materials and Methods

### Cell Lines and Culture

Colorectal cancer cell line (HCT-15) was kindly presented by Prof. Sugiyama, Department of Gastroenterology, Graduate School of Medical and Pharmaceutical Sciences, University of Toyama, Japan. Its BTG1 transfectants were prepared as previously done (14). They were maintained in RPMI 1640 medium supplemented with 10% fetal bovine serum (FBS), 100 units/ml penicillin, and 100 μg/ml streptomycin in a humidified atmosphere of 5% CO_2_ at 37°C. All cells were harvested by centrifugation, rinsed with phosphate buffer saline (PBS), and subjected to total protein and RNA extraction by homogenization.

### Cell Adhesion

To measure heterogeneous adhesion, 2 × 10^4^ cells were seeded in the fibronectin-coated wells and incubated in 5% CO_2_ at 37°C for 60, 90, and 120 min. After washing with PBS, the attaching cells were subjected to MTT assay. To determine homogeneous adhesion, 70% confluent cells were incubated with the same cells for 60, 90, and 120 min. Subsequently, the floating cells were subjected to MTT assay, and the adherent cells were calculated.

### Wound Healing Assay

Cells were seeded at a density of 1.0 × 10^6^ cells/well in 6-well culture plates. After they had grown to confluence, the cell monolayer was scraped with a pipette tip to create a scratch, washed by PBS for three times and cultured in the FBS-free medium. Cells were photographed at 0, 12, 24, and 48 h and the scratch area was measured using Image J.

### Transwell Chamber Assay

For invasion assay, cells were resuspended in serum-free RPMI 1640 and seeded in the Matrigel-coated insert on the top portion of the chamber (BD Bioscience). The lower compartment of the chamber contained 10% v/v FBS as a chemoattractant. After incubation at 37°C and 5% CO_2_ for 24 h, cells on the membrane were scrubbed, washed with PBS, fixed in 100% methanol, and stained with Giemsa dye for the measurement. For migration assay, the procedures were the same as mentioned above except for non-matrigel coating.

### Subjects and Pathology

Colorectal cancers (CRCs, n = 485) were collected from the surgical resection in the Affiliated Hospital of Kanagawa Cancer Center (Japan) between 1995 and 1999. The patients with CRC were 261 men and 224 women (26–85years, mean = 64.1 years). Among them, 207 cases are accompanied with lymph node metastasis and 28 cases with liver metastasis. None of the patients underwent chemotherapy, radiotherapy, or adjuvant before surgery. All of them provided written consent for use of tumor tissues for clinical research and the Ethical Committee of our hospital and Kanagawa Cancer Center approved the research protocol. We followed up the patients by consulting their case documents or by telephone.

All tissues were fixed in 10% neutral formalin, embedded in paraffin and cut into at 4 μm. These sections were stained by hematoxylin-and-eosin (HE) to confirm their histological characteristics. These tissues were subjected to the establishment of tissue microarrays under the guidance of HE staining. The staging for each colorectal cancer was evaluated according to the Union Internationale Contre le Cancer system (18). Histological architecture of CRCs was expressed in terms of WHO’s classification (19). Furthermore, tumor size, depth of invasion, lymphatic and venous invasion were determined.

### Immunohistochemistry

Consecutive sections of tissue microarrays (15) were deparaffinized with xylene, dehydrated with alcohol, and subjected to antigen retrieval by irradiating in target retrieval solution (TRS, DAKO, USA) for 15 min with microwave oven. The sections were quenched with 3% hydrogen peroxide in absolute methanol for 20 min to block endogenous peroxidase activity. Five percent of bovine serum albumin was then applied for 5 min to prevent non-specific binding. The sections were incubated with the rabbit antibody against BTG1 (1:100) from Proteintech for 15 min, then treated with the anti-rabbit conjugated to horseradish peroxidase (DAKO, USA) antibodies for 15 min. All the incubations were performed in a microwave oven to allow intermittent irradiation as described previously (14). Binding sites were visualized with diaminobenzidine. After counterstaining with Mayer’s hematoxylin, the sections were dehydrated, cleared, and mounted. Omission of the primary antibody was used as a negative control.

BTG1 protein was positively localized in the cytoplasm. One hundred cells were randomly selected and counted from five representative fields of each section blindly by two independent observers (Zhao S and Zheng HC). The positive percentage of counted cells was graded semi-quantitatively with a four-scale scheme: negative (−), less than 5%; weakly positive (+), 6–25%; moderately positive (++), 26–50%; and strongly positive (+++), more than 51%.

### The Measurement of Carcinoembroyonic Antigen and Cancer Antigen 19-9

Serum CEA was determined using Chemiluminescence Immunoassay (Diagnostic Agnostic Automation Inc.). Briefly, 50 μl of standard (0–120 ng/ml), specimens (serum and cell culture supernatant), and controls was dispensed into appropriate wells. Then, we added 100 μl of Enzyme Conjugate Reagent into each well, gently mixed, and incubated the plate at room temperature for 60 min. The microtiter wells were rinsed and flicked with wash buffer. After that, residual water droplets were removed by striking the well sharply onto absorbent paper. Finally, 100 μl chemiluminescence substrate solution was dispensed into each well, mixed gently, and subjected to absorbance determination.

Serum CA19-9 was measured using Chemiluminescence Immunoassay (CLIA) Diagnostic Test Kit (Jei Daniel Biotech Corp.). In brief, 50 μl of standard, specimens, and controls was dispensed into the appropriate wells. After that, 50 μl of CA 19-9-conjugate solution was added into each well, followed by thoroughly mixing for 30 s. The plate was subjected to incubation at 37–40°C for 15 min. We removed the incubation mixture by flicking the plate content into a waste container, rinsing, and flicking the microtiter wells five times with working wash solution. Finally, 50 µl of working substrate solution was added into all wells, which was subsequently incubated at room temperature for 5 min and read with a chemiluminescence microtiter plate reader.

### Western Blot

Denatured protein was separated on an SDS-polyacrylamide gel (10% acrylamide) and transferred to a Hybond membrane (Amersham), which was then blocked overnight in 5% skim milk in TBST (10 mmol/L Tris-HCl, 150 mmol/L NaCl, 0.1% Tween 20). For immunoblotting, the membrane was incubated for 1 h with the primary antibody (**Table 1**). Then, it was rinsed with TBST and incubated with anti-rabbit or anti-mouse IgG conjugated to horseradish peroxidase (DAKO, USA, 1:1,000) for 1 h. Bands were visualized by ECL-Plus detection reagents (Santa Cruz). Densitometric quantification was performed with *β*-actin as an internal control using Scion Image.

**Table 1:** Primers’ design for RT-PCR.

Names	Primer’s sequence	Distribution	AT(°C)	Productsize (bp)	Extensiontime (s)
*Zeb1*	F: 5′-GCTTGTGATTTGTGTGACAAGA-3′ R: 5′-AATCGCATGTGTTCAATCAA- 3′	XM_017016603.1 6160-6305	60	146	34
*Vimentin*	F: 5′-GGCGATGGCCCAGCTGTAAG-3′ R: 5′-CTGCTGTCCCGCCGATTGAG-3’	NM_003380.3 131-261	60	131	34
*CEA*	F:5′-AGCCTCACTTCTAAACTTCT-3′R:5′-TCCCCTTTGTACCAGCTGTA-3′	NM_001277163151-300	50	150	34
*GAPDH*	F: 5′-CAATGACCCCTTCATTGACC-3′ R: 5′-TGGAAGATGGTGATGGGATT-3′	NM_ 002046.3 201-335	60	135	34

AT, annealing temperature.

### RT-PCR

Total RNA was extracted from cells using QIAGEN RNeasy mini kit (Hilden, Germany). Two micrograms of total RNA was subjected to cDNA synthesis using AMV reverse transcriptase and random primer (Takara, Japan). According to Genbank, oligonucleotide primers were designed and shown in **Table 2**. Real-time PCR was performed according to the protocol of SYBR Premix Ex Taq™ II kit (Takara).

**Table 2:** Antibodies used for Western blot.

Name	Source	Company
N-cadherin	Rabbit	Wanleibio
E-cadherin	Rabbit	Wanleibio
Twist	Rabbit	Wanleibio
WAVE2 (C-6)	Mouse	Santa Cruz Biotechnology
CEA (161)	Mouse	Santa Cruz Biotechnology
c-jun(B-1)	Mouse	Santa Cruz Biotechnology
PI3K	Rabbit	Abcam
MMP-9	Rabbilt	Santa Cruz
*β*-actin	Mouse	Santa Cruz

### Bioinformatics Analysis

The trancriptomic and clinicopathological data of colorectal cancer patients were downloaded from the TCGA database by TCGA-assembler of the R software. We analyzed BTG1 mRNA level between colorectal normal and cancer tissues using Oncomine and TCGA data. *BTG1* expression was compared with clinicopathological and prognostic data of colorectal cancer patients. GSEA was performed with GSEA-3.0. We used *BTG1* level as a phenotype label and analyzed pathway enrichment. Kaplan–Meier plotter was employed to analyze the prognostic significance of BTG1 mRNA and methylation.

### Statistical Analysis

Statistical evaluation was performed using *Spearman’s* correlation test to analyze the rank data and student t test to compare the means. *Kaplan*–*Meier* survival plots were generated, and comparisons between survival curves were made with the log-rank statistics. *Cox*’s proportional hazards model was employed for multivariate analysis. SPSS 10.0 software was applied to analyze all data, and *P* < 0.05 was considered statistically significant.

## Results

### The Clinicopathological and Prognostic Significances of BTG1 Methylation or Expression in Colorectal Cancer

**Figure 1:**
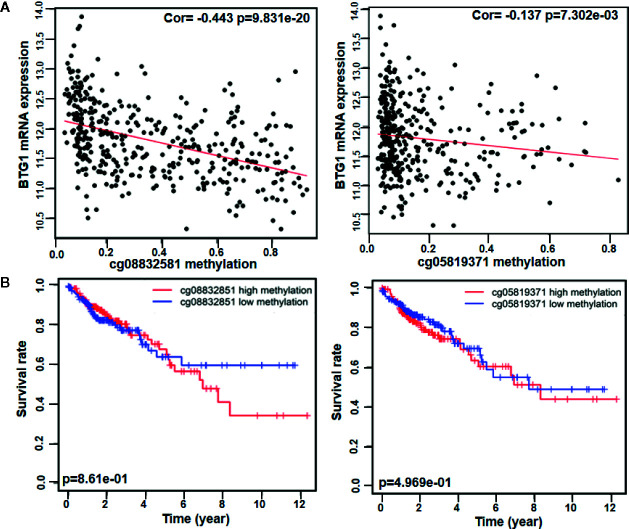
The prognostic significance of *BTG1* methylation in colorectal cancer. There was negative relationship between *BTG1* methylation and mRNA expression (**A**, *P* < 0.05) and between *BTG1* hypermethylation and prognosis in the colorectal cancers (**B**, *P* < 0.05).

As indicated in **Table 3** and **Figure 1A**, *BTG1* mRNA expression was negatively correlated with its promoter methylation in colorectal cancer (P < 0.05). However, *BTG1* hypermethylation at cg05819371 and cg08832851 was significantly related with poor prognosis of the colorectal cancer patients (**Figure 1B** *, P* < *0.05*).

**Table 3:** The correlation between *BTG1* methylation and mRNA expression in colorectal cancer.

Methylation site	Pearson correlation coefficient	P value
cg04100724	0.064	2.166e−01
cg04211745	−0.017	7.371e−01
cg05819371	−0.137	7.302e−03
cg06551025	−0.018	7.224e−01
cg08832851	−0.043	9.831e−20
cg09918929	−0.021	6.852e−01
cg13132650	−0.049	3.441e−01
cg20078640	0.02	6.938e−01
cg21381360	0.041	4.259e−01
cg25218905	0.043	4.308e−01

**Figure 2:**
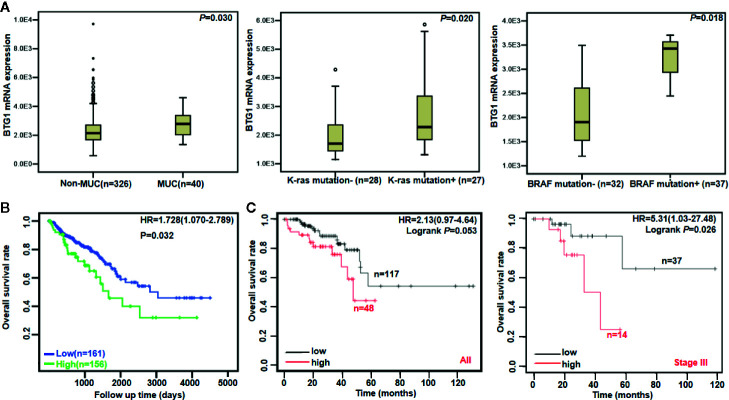
The clinicopathological and prognostic significances of *BTG1* mRNA in colorectal cancer. TCGA database showed that *BTG1* was lower expressed in well-, moderately, and poorly differentiated (non-MUC) than in mucinous (MUC) adenocarcinomas (**A**, *P* < 0.05). *BTG1* expression was positively correlated with K-ras and BRAF mutation of colorectal cancers (**A**, *P* < 0.05). *BTG1* expression was negatively related to overall survival rate of the patients with colorectal cancer (**B**, *P* < 0.05). The prognostic significance of *BTG1* mRNA expression was also analyzed using Kaplan–Meier plotter database **(C)**. HR, hazard ratio.

There was no difference in *BTG1* mRNA expression between colorectal cancer and normal tissues using Oncomine database (P > 0.05, data not shown). In TCGA data, *BTG1* mRNA expression was lower in well-, moderately, and poorly differentiated than in mucinous adenocarcinomas (**Figure 2A**, P < 0.05). It was positively correlated with K-ras mutation and BRAF mutation of colorectal cancer (**Figure 2A**, P < 0.05). Additionally, Kaplan–Meier analysis showed the negative correlation between *BTG1* mRNA expression and overall survival rate of all cancer patients (**Figure 2B**, *P* < 0.05). According to the Kaplan–Meier plotter, *BTG1* mRNA expression *was* negatively *correlated with* the overall survival rate of Stage-III cancer patients (**Figure 2C**, *P* < 0.05). Cox’s hazard proportional analysis indicated that younger age and distant metastasis were independent factors for the adverse prognosis of the patients with colorectal cancer (**Table 4**, *P* < 0.05).

**Table 4:** Survival analysis of hazard factors of the prognosis of the patients with colorectal cancer.

Parameters	Multivariate analysis	
	HR(95%CI)	P value
Age(>60year)	2.601(1.443–4.688)	0.001
Gender (female)	0.892(0.572–1.391)	0.615
Cancer type (colon *vs* rectum)	0.635(0.318–1.267)	0.198
T(T_3–4_)	2.069(0.801–5.346)	0.133
N(+)	0.636(0.187–2.162)	0.469
M(+)	2.980(1.715–5.175)	<0.001
Stage (II–IV)	3.330(0.861–12.877)	0.081
*BTG1* mRNA expression (high)	1.293(0.824–2.030)	0.263

HR, hazard ratio, T, T staging, M, metastasis staging, N, lymph node metastasis.

As summarized in **Table 5**, we conducted a GSEA to analyze *BTG1*-related signal pathways in colorectal cancer. The enriched pathways included cytokine–cytokine receptor interaction, regulation of autophagy, leukocyte transendothelial migration, natural killer cell-mediated cytotoxicity, Toll-like-receptor signaling pathway, chemokine signaling pathway, and so on (*P* < 0.05).

**Table 5:** BTG1-related signal pathways in colorectal cancer.

Names	Size	P value
Cytokine–cytokine receptor interaction	234	<0.001
Regulation of autophagy	21	<0.001
Leukocyte transendothelial migration	111	<0.001
Natural killer cell-mediated cytotoxicity	116	<0.001
Toll-like-receptor signaling pathway	87	<0.001
Chemokine signaling pathway	183	<0.001
Long term potentiation	66	<0.001
Aldosterone related sodium reabsorption	40	<0.001

### The Relationship Between BTG1 Expression Clinicopathological Parameters in Colorectal Cancer

**Figure 3:**
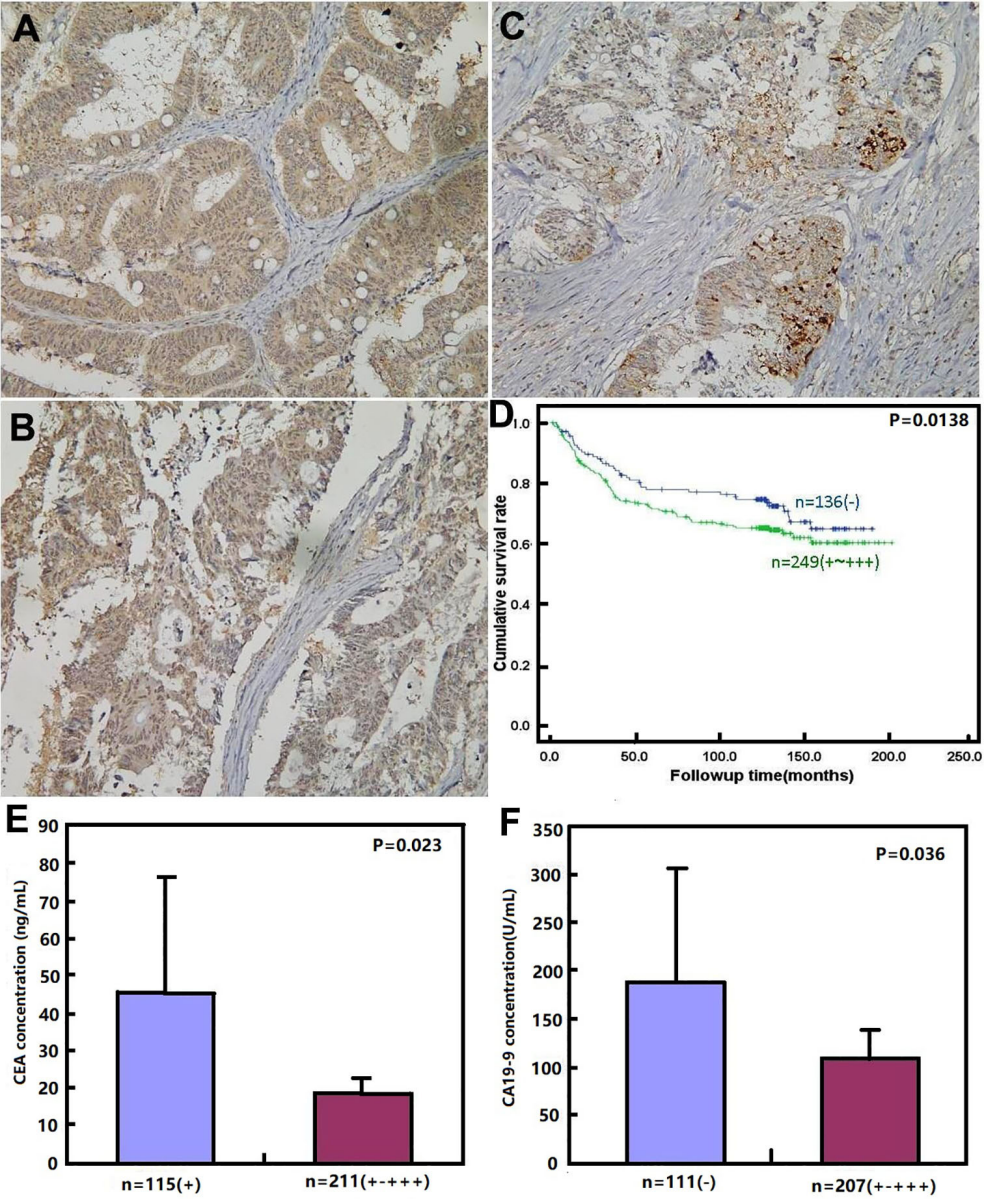
BTG1 protein expression and its prognostic significance in colorectal cancer. BTG1 protein was positively detected in the cytoplasm of colorectal cancers **(A**–**C)**. Kaplan–Meier analysis showed no relationship between BTG1 protein expression and the cumulative survival rate of the patients with colorectal cancer (*P* > 0.05), **(D)**. There was negative association of BTG1 expression with serum CEA **(E)** and CA19-9 **(F)** levels (*P* < 0.05).

As shown in **Figures 3A–C** and **Table 6**, BTG1 expression was positively related to depth of invasion, venous invasion, lymph node metastasis, distant metastasis, and TNM staging (*P* < 0.05) but not correlated with age, sex, lymphatic invasion, liver metastasis, or differentiation of colorectal cancer (*P* > 0.05). Follow-up information was available on 385 colorectal cancer patients for periods ranging from 1.6 months to 16.8 years (median = 10.5 years). Survival curves for colorectal cancers were stratified according to BTG1 expression (**Figure 3D**). Univariate analysis using Kaplan–Meier method indicated no correlation between BTG1 expression and cumulative survival rate of patients with colorectal cancer despite stratification to depth of invasion (*P* > 0.05). The patients with BTG1-positive cancer showed lower serum level of CEA and CA19-9 than those with BTG1-negative cancer (*P* < 0.05, **Figures 3E, F** **)**. Multivariate analysis using Cox’s proportional hazard model indicated that depth of invasion and distant metastasis (*P* < 0.05) but not age, sex, lymphatic and venous invasion, lymph node metastasis, liver metastasis, differentiation; TNM staging and BTG1 expression (**Table 7**, *P* > 0.05) were independent prognostic factors for overall colorectal cancer patients.

**Table 6:** Relationship between BTG1 expression and clinicopathological features of colorectal cancer.

Clinicopathologicalfeatures	n	BTG1 expression					
		−	+	++	+++	PR (%)	P value
Age(years)							0.812
<65	280	95	53	76	56	66.1	
≥65	205	72	40	51	42	64.9	
Sex							0.981
Male	261	88	51	72	50	66.3	
Female	224	79	42	55	48	64.7	
Depth of invasion							<0.001
T_is_–T_2_	126	57	27	28	14	54.8	
T_3_–T_4_	338	104	62	93	79	69.2	
Lymphatic invasion							0.146
−	197	75	40	52	30	61.9	
+	207	71	36	55	45	65.7	
Venous invasion							0.003
−	195	78	42	47	28	60.0	
+	196	59	33	57	47	69.9	
Lymph node metastasis							0.019
−	259	97	53	65	44	62.5	
+	207	62	37	57	51	70.0	
Liver metastasis							0.090
−	450	158	88	117	87	64.9	
+	28	7	4	8	9	75.0	
Distant metastasis							0.041
−	445	158	85	116	86	64.5	
+	36	7	8	10	11	80.6	
TNM staging							0.002
0–II	247	95	53	60	39	61.5	
III–IV	215	62	37	60	56	71.2	
Differentiation							0.193
Well-differentiated	197	72	43	47	35	63.5	
Moderately differentiated	231	72	37	71	51	68.8	
Poorly differentiated	32	14	4	7	7	56.3	

PR, positive rate; T_is_, carcinoma in situ; T_1_, lamina propria and submucosa; T_2_, muscularis propria; T_3_, subserosa and exposure to serosa_;_ T_4_, invade other organs or perforate visceral peritoneum; TNM, tumor-node-metastasis.

**Table 7:** Multivariate analysis of clinicopathological variables for the survival of the patients with colorectal cancer.

Clinicopathological parameters	Relative risk (95%CI)	P value
Age (≥65years)	0.775(0.492–1.222)	0.273
Sex (female)	0.884(0.558–1.401)	0.600
Depth of invasion (T_2–4_)	6.142(2.140–17.626)	0.001
Lymphatic invasion (+)	1.145(0.684–1.917)	0.607
Venous invasion (+)	1.016(0.598–1.726)	0.952
Lymph node metastasis (+)	1.515(0.452–5.075)	0.500
Liver metastasis (+)	0.310(0.063–1.533)	0.151
Distant metastasis (+)	13.189(2.886-60.272)	0.001
TNM staging (III-IV)	2.071(0.543–7.903)	0.287
Differentiation (poorly differentiated)	1.109(0.772–1.595)	0.575
BTG1 expression (+++)	1.105(0.676–1.808)	0.691

CI, confidence interval; TNM, tumor-node-metastasis.

### The Effects of BTG1 Expression on Aggressive Phenotypes of Colorectal Cancer Cells

**Figure 4:**
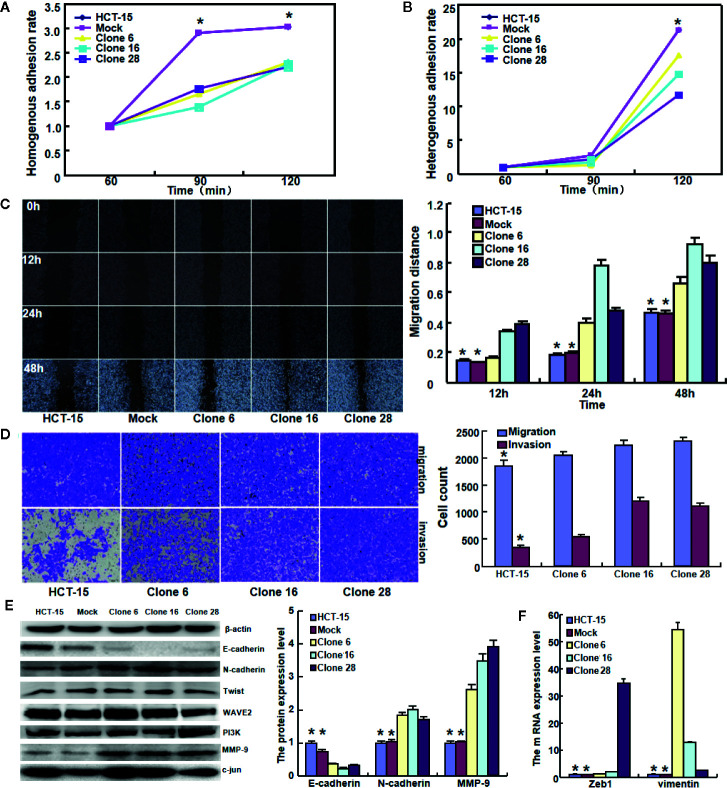
The effects of *BTG1* expression on adhesion, migration, and invasion of colorectal cancer cells. BTG1-overexpressing HCT-15 had a lower homogenous **(A)** and heterogeneous **(B)** adhesion, a stronger ability to migrate and invade by wound healing **(C)** and Transwell assays **(D)**. The phenotype-related molecules were screened by Western blot **(E)** and real-time RT-PCR **(F)**. Results are representative of three different experiments, and data are expressed as mean ± SE. *P < 0.05, compared with the transfectants.

**Figure 5:**
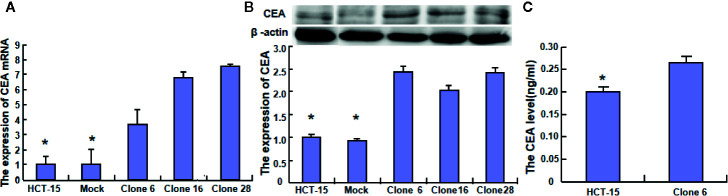
The effects of *BTG1* on CEA expression and secretion in colorectal cancer cells. The CEA expression was examined in HCT-15 and its BTG1 transfectants by real-time RT-PCR **(A)** and Western blot **(B)**. CEA secretion was also determined by ELISA **(C)**. Results are representative of three different experiments, and data are expressed as mean ± SE. *P < 0.05, compared with the transfectants.

To clarify the role of *BTG1*, its expressing plasmid was successfully transfected into HCT-15 as previously performed (14). The transfectants showed a lower homogenous and heterogeneous adhesion, higher migration and invasion than the mock and control (**Figures 4A–D**, *P* < 0.05). Additionally, BTG1 transfectants displayed E-cadherin hypoexpression, N-cadherin and MMP-9 hyperexpression, compared with the control and mock cells by Western blot (**Figure 4E**, *P* < 0.05), but there was no difference in the expression of Twist, WAVE2, PI3K, and c-jun between the transfectants, mock and control cells (*P* > 0.05). According to real-time RT-PCR, *Zeb1* and *Vimentin* mRNA was increased in BTG1 transfectants in comparison to the control and mock (**Figure 4F**, *P* < 0.05). To clarify the negative correlation between serum CEA level and BTG1 expression, we examined CEA expression and secretion and found that both indicators were higher in *BTG1*-overexpressing HCT-15 cells than in the control or mock by real-time RT-PCR, Western blot, and ELISA (**Figures 5A–C**, *P* < 0.05).

## Discussion

BTG1 expression was restored after 5-aza-2′-deoxycytidine treatment in gastric, breast, and ovarian cancer cells, indicating that BTG1 promoter methylation accounted for its down-regulated expression in cancer cells (15, 20, 21). Jung et al. (22) found that *BTG1* expression was lower in colorectal cancer than in control and in metastatic than in primary cancer due to the hypermethylation of *BTG1* promoter. Here, our finding demonstrated a negative relationship between *BTG1* mRNA expression and promoter methylation but only a close relationship between *BTG1*. hypermethylation of cg05819371 and cg08832851 sites and poor prognosis of the colorectal cancer patients

In line with the data about gastric cancer (15), BTG1 expression was positively linked to aggressive features of colorectal cancer, including depth of invasion, venous invasion, lymph node metastasis, and TNM staging. Its mRNA expression was also associated with k-ras and BRAF mutation in colorectal cancer. According to the literature (14), colorectal metastatic cancer in lymph node showed more BTG1 expression than in primary cancer. In contrast, a body of evidence indicated that BTG1 expression was negatively correlated with tumor invasion, lymph node metastasis, clinic stage, histological grade, pathological differentiation, or a low survival rate of patients with hepatocellular, thyroid, breast, or esophageal cancer (23–26). Chen et al. (27) also reported that BTG1 expression was higher in prostate cancer cell line LNCaP than its aggressively metastatic, AIC4-2 at both mRNA and protein levels. Kanda et al. (28) demonstrated that BTG1 down-regulation led to adverse prognosis, specifically in patients with proximal non-diffuse and diffuse gastric cancers. Taken together, it was suggested that BTG1 expression might be involved in the progression of colorectal cancer and be considered as a good marker to indicate the aggressive behaviors of colorectal cancer.

BTG1 hypoexpression was reported to significantly correlate with aggressive features and worse prognosis of thyroid cancer (23), esophageal cancer (26), breast cancer (24), non-small cell lung cancer (29), and skin squamous carcinoma (30). Zhang et al. (31) found that the cumulative survival rate of BTG1-positive patients was significantly higher than that of BTG1-negative patients with colon cancer as an independent factor. Kamalakaran et al. (32) identified differential methylation of CpG islands proximal to BTG1 as having prognostic value independent of subtypes and other clinical factors of luminal breast cancers. Down-regulated *BTG1* mRNA expression in gastric cancer and hepatocelluar carcinoma was significantly associated with shorter disease-specific and recurrence-free survival as an independent prognostic factor (28, 33). However, BTG1 protein expression was positively associated with poor prognosis of gastric cancer (15), in agreement with our data of *BTG1* mRNA expression according to TCGA databases. These findings paralleled with the positive association between BTG1 protein expression and aggressive behaviors of colorectal cancer.

To clarify the effects of BTG1 on the invasion and metastasis of colorectal cancer cells, we overexpressed it in HCT-15 cells and found that BTG1 weakened adhesion, but enhanced the ability to migrate and invade. Various studies showed that BTG1 overexpression suppressed proliferation, migration and invasion, and induced apoptosis and cell cycle arrest of lung, kidney, breast, esophageal, hepatocellular, nasopharyngeal, and thyroid cancers with Cyclin D1, Bcl-2, and MMP-9 hypoexpression (23–26, 29, 34, 35). BTG1 overexpression was observed to inhibit migration and invasion of breast cancer cells with MMP-2 and -9 hypoexpression and E-cadherin hyperexpression (36). Our previous work also showed that BTG1 overexpression might *in vivo* and *in vitro* reverse the aggressive phenotypes of colorectal cancer cells (14). Epithelial–mesenchymal transition (EMT) is a process that epithelial cells are converted from a phenotypic shift from cells with tight cell–cell junctions, clear basal and apical polarity (epithelial markers: E-cadherin and plakoglobin), and sheet-like growth architecture into spindle-like and motile cells (mesenchymal markers: N-cadherin, vimentin, Zeb, and Twist) (37, 38). Mao et al. (39) found that BTG3 expression significantly inhibited cell growth, migration, invasion, and EMT of colorectal cancer cells *via* Wnt/*β*-catenin signaling pathway. Here, BTG1 overexpression resulted in stronger migration and invasion with E-cadherin hypoexpression, N-cadherin, Vimentin and Zeb1 hyperexpression, indicating that BTG1 might promote invasion and metastasis by inducing EMT. However, we achieved the opposite results in HCT-116 cells although they were isolated in the same male colorectal cancer patient with Duke’s C staging. Certainly, we also can’t exclude the special situation in HCT-15 cells because both cell lines derive from different clones. Therefore, we should be careful to employ BTG1 for gene therapy target.

Carcino-embryonic antigen (CEA) is a glycosyl phosphatidyl inositol (GPI)-anchored membrane glycoprotein and acts as L-selectin and E-selectin ligands for cellular adhesion. It was found that serum from individuals with gastric cancer, pancreatic cancer, lung cancer, breast cancer, and medullary thyroid carcinoma had higher levels of CEA than healthy individuals. In colorectal cancer, CEA might be considered as a potential marker to predict early progression and worse prognosis (40–42). Here, there appeared a negative association between BTG1 expression and serum CEA level. In contrast, BTG1 overexpression increased CEA expression and secretion, which was closely linked to a strong ability of cancer cells to migrate and invade. In colorectal cancer cells, BTG1 might suppress proliferation and tumor growth and induce apoptosis and differentiation [11], which resulted in a higher anchoring of CEA to membrane, a low necrosis and subsequent release of CEA into the blood vessel (43). However, it can’t be explained why BTG1 weakened the ability of HCT-15 cells to homogeneously and heterogeneously adhere. According to our knowledge, it needs further investigation about how BTG1 regulates CEA expression and its biological functions in malignancies.

In summary, our study indicated that BTG1 expression might be positively linked to invasion, metastasis, and poor prognosis of colorectal cancers. BTG1 overexpression might reduce adhesion, and promote invasion and metastasis by inducing epithelial–mesenchymal transition. BTG1 expression should be employed to indicate the aggressive behaviors and worse prognosis of colorectal cancer.

## Data Availability Statement

The raw data supporting the conclusions of this article will be made available by the authors, without undue reservation.

## Ethics Statement

The studies involving human participants were reviewed and approved by the Kanagawa Cancer Center, Affiliated Hospital of Chengde Medical University. Written informed consent to participate in this study was provided by the participants’ legal guardian/next of kin.

## Author Contributions

SZ, HX, C-LH, H-MJ, and H-CZ designed and carried out the experiments, and H-CZ wrote the draft. All authors contributed to the article and approved the submitted version.

## Funding

This study was supported by the Award for Liaoning Distinguished Professor and National Natural Scientific Foundation of China (Grant no.81672700).

## Conflict of Interest

The authors declare that the research was conducted in the absence of any commercial or financial relationships that could be construed as a potential conflict of interest.
